# A qualitative exploration of participants’ perspectives and experiences of novel digital health infrastructure to enhance patient care in remote communities within the Home Health Project

**DOI:** 10.1371/journal.pdig.0000600

**Published:** 2024-11-01

**Authors:** Madeleine Kearney, Leona Ryan, Rory Coyne, Hemendra Worlikar, Ian McCabe, Jennifer Doran, Peter J. Carr, Jack Pinder, Seán Coleman, Cornelia Connolly, Jane C. Walsh, Derek O’Keeffe

**Affiliations:** 1 School of Psychology, University of Galway, Galway, Ireland; 2 Health Innovation Via Engineering Laboratory, University of Galway, Galway, Ireland; 3 School of Medicine, University of Galway, Galway, Ireland; 4 CÚRAM, SFI Research Centre for Medical Devices, University of Galway, Galway, Ireland; 5 School of Nursing and Midwifery, University of Galway, Galway, Ireland; 6 School of Education, University of Galway, Galway, Ireland; Iran University of Medical Sciences, ISLAMIC REPUBLIC OF IRAN

## Abstract

The Home Health Project, set on Clare Island, five kilometres off the Irish Atlantic coast, is a pilot exploration of ways in which various forms of technology can be utilised to improve healthcare for individuals living in isolated communities. The integration of digital health technologies presents enormous potential to revolutionise the accessibility of healthcare systems for those living in remote communities, allowing patient care to function outside of traditional healthcare settings. This study aims to explore the personal experiences and perspectives of participants who are using digital technologies in the delivery of their healthcare as part of the Home Health Project. Individual semi-structured interviews were conducted with nine members of the Clare Island community participating in the Home Health Project. Interviews took place in-person, in June 2023. Interviews were audio-recorded and transcribed verbatim. The data were analysed inductively using reflexive thematic analysis. To identify determinants of engagement with the Home Health Project, the data was then deductively coded to the Theoretical Domains Framework (TDF) and organised into themes. Seven of the possible 14 TDF domains were supported by the interview data as influences on engagement with the Project: Knowledge, Beliefs about capabilities, Optimism, Intentions, Environmental context and resources, Social influences and Emotion. Overall, participants evaluated the Home Health Project as being of high quality which contributed to self-reported increases in health literacy, autonomy, and feeling well supported in having their health concerns addressed. There was some apprehension related to data protection, coupled with a desire for extended training to address aspects of digital illiteracy. Future iterations can capitalise on the findings of this study by refining the technologies to reflect tailored health information, personalised to the individual user.

## Author summary

The Clare Island Home Health Project aims to deliver virtual healthcare using a combination of video consultations, remote physiological monitoring, and artificial intelligence to members of a remote island community off the west coast of Ireland. The project seeks to improve patient care by assisting with the management of chronic disease. In this study, nine participants in the Project were interviewed to gather insights on their experiences with the forms of technology that are used during the Project. Thematic analysis was used to generate themes from the interview data, to identify recurring patterns of expression among participants in relation to their experiences. The findings were then mapped onto the Theoretical Domains Framework to identify the factors influencing participants’ level of engagement with the technology. The findings revealed that factors such as optimism, social influences and emotion were influential in determining the extent to which participants engaged with the technology. Perception of the Project was positive overall; participants felt that the Project was contributing to an improved sense of autonomy and ability to manage and understand their health-related concerns. The findings of this study can be applied to efforts to improve digital health technology by personalising the technology to the user.

## 1. Introduction

Digital health technologies are effective when used in healthcare to promote early detection and prevention of disease using remote patient monitoring [1]. Technology permits increased patient empowerment through the use of self-management tools which facilitate patients to engage in self-monitoring and to be more actively engaged in their healthcare [2,3]. Digital health interventions are revolutionising healthcare, particularly for those living in remote communities, through allowing patient care to be delivered outside of traditional healthcare settings [4]. Remote island communities are presented with numerous challenges in accessing equitable healthcare compared to urban areas, such as limited availability of local services due to geographical restrictions and unpredictable weather conditions [5].

In keeping with the World Health Organisation’s (WHO) “Health for All” initiative [6], delivering universal health coverage to isolated communities becomes particularly crucial. Achieving this aim in these settings necessitates innovative strategies to overcome geographical barriers and ensure equitable access to healthcare services that promote overall well-being. Digital health solutions have the potential to help bridge the gap of inequity and overcome longstanding geographical barriers to enhance healthcare for island populations and contribute to improved health outcomes [7].

Health policymakers, clinicians and researchers are striving to improve the quality of healthcare through the application of innovative interventions that aim to encourage more effective, efficient, accessible, and patient-focused services [8]. The Medical Research Council recommends the utilisation of theory to support interventions which promote the implementation of evidence-based practices [9]. However, previous research has shown that these interventions rarely report the use of underpinning theory to evaluate the design and implementation of healthcare interventions, making replication of successful interventions challenging [10,11]. There is a need for a more explicit use of theory to inform implementation of health interventions through understanding mechanisms of change and identifying specific factors that could influence the adoption of an intervention [12].

Innovative digital health technologies offer unique opportunities to gather data on behaviour patterns and intervene with targeted interventions in real-time [13]. However, to fully realise their potential, the implementation of new healthcare practices or changing existing practices within a population will require both individual and collective behaviour change [12]. Adhering to established frameworks for behaviour change provides a valuable tool for evaluating and optimising the effectiveness of digital health interventions. Health behaviour change theories offer a robust mechanism for understanding psychosocial and structural processes behind behaviour change and regulation. Health behaviour change interventions underpinned with psychological theory are reportedly more effective than those that are not [14,15] and can result in more significant health behaviour changes [16], as well as overall greater intervention success [17].

The Theoretical Domains Framework (TDF) is an overarching framework that was developed by a team of behavioural scientists and implementation specialists to understand the influences on health behaviour change and to inform evidence-based recommendations regarding the implementation of behaviour change interventions [18]. The TDF was developed through a synthesis of 128 constructs from 33 theories of behaviour change and contains 14 domains which encompass the conceptual determinants of human behaviour and stimulants of behaviour change [19,20]. Rather than being a theory, the TDF is a theoretical framework. As such, it provides a theoretical lens through which to view affective, social, cognitive, and environmental influences on behaviour. Another strength of this theoretical framework is that it accounts for the barriers and facilitators of the uptake of health interventions within specific contexts [8]. This theoretical framework has been applied widely within the health sciences to address a range of objectives, including identifying influences on behaviour, systematically designing interventions, and evaluating randomised trials [12]. As such, this approach is well-suited to identifying the determinants of engagement with novel digital health interventions such as the Clare Island Home Health Project.

The Behaviour Change Wheel (BCW) is a systematic framework designed to guide the development of effective behaviour change interventions [17]. The COM-B model exists within the hub of the BCW and proposes that there are three interdependent preconditions necessary for a behaviour to occur: Capability (physical and psychological), Opportunity (social and physical) and Motivation (automatic and reflective). Moving outwards from the COM-B core, each behavioural component is analysed in greater detail within the TDF, which details specific mechanisms of change to facilitate behaviour change [12]. The two outermost rings include intervention functions, which contain active mechanisms that modify behaviour, and policy categories which details various supports for behavioural change on an organisational, societal and community level [17].

The application of the TDF has informed the design and implementation of interventions based around many health behaviours, including dietary habits [21] and physical activity [22]. A review investigating the application of the TDF within health behaviour interventions to inform future development found that there is limited evidence of the framework being used to support health interventions [23]. Specifically, only three of the ten included studies used the TDF [24–26]. Therefore, the explanatory potential of the TDF to inform the delivery of health behaviour change interventions remains largely unstudied.

**Fig 1:**
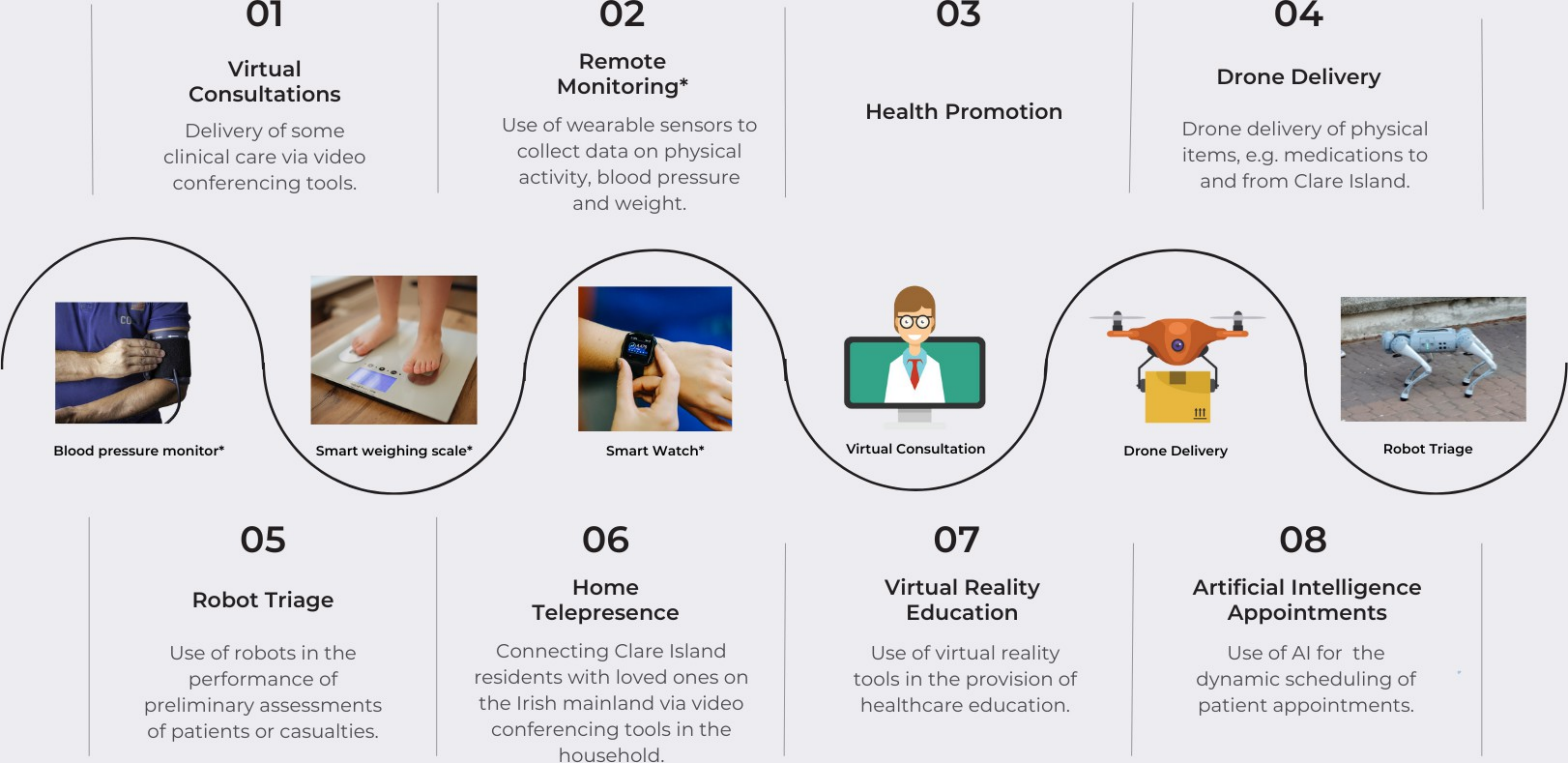
Overview of the work packages and novel technologies that comprise the Clare Island Home Health Project. The data collection tools that are being used as part of Work Package 2 (remote monitoring) are indicated using an asterisk (*). “Fitbit Versa” by Kārlis Dambrāns is licensed under CC BY 2.0. “Girl stands and weighs herself on white modern electronic smart scale” by Nenad Stojkovic is licensed under CC BY 2.0.

This study is nested within the larger Home Health Project. This Project addresses the unique challenges of delivering healthcare to isolated communities in Ireland. It focuses on islands like Clare Island (an island situated off the west coast of Ireland, with only 165 inhabitants and a population density of only 30 people per square kilometre) as well as others such as Inis Turk, and Inis Boffin. By harnessing telemedicine and pioneering a ‘virtual ward’ model, it aims to improve access to care, empower patients, and alleviate strain on the healthcare system. The Project envisions remote consultations and telemonitoring of chronic conditions, enhancing patients’ experience and engagement with their health management. This will be facilitated through the collection of longitudinal data regarding blood pressure, physical activity and weight, using remote monitoring devices (smart watches, blood pressure cuffs, smart weighing scales). Using artificial intelligence (AI), this data will then inform timely clinical decision-making for the management of conditions such as hypertension. Fig 1 provides a visual summary of the Home Health Project, including the work packages and data collection tools involved. Readers are also directed to Connolly et al. [27] for further information.

The Home Health Project, funded by Science Foundation Ireland, is a pilot exploration of how various forms of technology can be leveraged to improve healthcare for individuals living in rural communities [27]. This Project is based in two neighbouring communities in County Mayo, Ireland. The community of Louisburgh, located on the mainland, will continue to receive normal care through their General Practitioner (GP). The second participant group, the remote island community of Clare Island, will trial a suite of state-of the-art medical technologies, including wearable exercise trackers with associated goals, remote blood pressure monitoring, virtual consultations with clinicians, and remotely delivered nutritional advice. This suite of interventions aims to deliver next-generation remote healthcare and chronic disease management in the community [27]. This community-based intervention is focused on improving the overall cardiovascular health of participants. The expected benefits include better management of hypertension, increased physical activity, better eating habits, reduced healthcare costs and an overall improved outlook on cardiovascular health. This Project, the first of its kind globally, aims to improve the management of patient care for the 165 residents on Clare Island and make the island a leading example for the delivery of digital health solutions.

The implementation of such a futuristic, ground-breaking suite of interventions will not come without challenges. However, as technology will play a vital role in the future of healthcare it is imperative that the opportunity is taken to gain an insight into the first-hand experiences of the Home Health Project participants’ use of digital technologies. In doing so, a deeper understanding of barriers and facilitators to the success of similar interventions in the future can be gained. This study therefore aims to investigate the implementation of the Home Health Project on Clare Island by gathering insights into the personal experiences of Project participants. This overall aim was achieved through the following three objectives:

1. To qualitatively explore attitudes and perspectives of islands towards the novel technologies within the Home Health Project.
2. To qualitatively explore participants’ level of engagement and perceived ability to engage with the novel technologies within the Home Health Project.
3. To identify the determinants of engagement with the Home Health Project through mapping the codes generated by a reflexive thematic analysis onto the Theoretical Domains Framework (TDF).

## 2. Method

The reporting of the study is in accordance with the consolidated criteria for reporting qualitative research (COREQ) checklist [28]. Deviations from the checklist occur where the construct was not compatible with the assumptions or methodological approach that underpins our analytical method, reflexive thematic analysis (RTA). To retain the fidelity of the methodological approach taken, ‘data saturation’ is replaced with ‘information power’ where the collection of rich, meaningful data is prioritised over quantity of respondents [29,30]. This study adopted the concept of information power, as described by Malterud et al. [30], to guide participant selection rather than solely relying on data saturation. Saturation aims to gather a sufficient amount of data to cover the topic, but it does not ensure rich or in-depth understanding. Information power, in contrast, prioritises the quality and depth of information gleaned from participants. Here, reaching a point of information power signifies that the data offers enough insightful and meaningful details to address the research questions.

This approach offers several advantages, including increased efficiency and a more nuanced exploration of the phenomenon under study [29]. Furthermore, information power aligns well with the characteristics of this particular research project. The narrow aim of the study (1), the specific sample population (2), the application of established theory (3), the focus on strong quality of dialogue during data collection (4), and the planned in-depth analysis strategy (5) all contribute to achieving information power with a strategically selected sample size [30]. This targeted recruitment and flexible approach to sample size determination aim to maximize the quality of the data collected [29]. In addition, to maintain methodological integrity, participant validation was not feasible due to the researcher’s central role in the interpretation of the data and the potential for participant perspectives to evolve over time [31].

### 2.1. Research team and reflexivity

The research team is comprised of authors with backgrounds in medicine (JD, DOK, SC), nursing (PJC), engineering (HW, DOK, IMcC), education (CC), advocacy (JP), and health psychology (JW, LR, RC, MK). The lead researcher (MK) conducted this research as part of their Stage 1 Health Psychology training, and as such has received substantial training on quantitative and experimental research methodology. Such research paradigms are largely informed by (post)positivist epistemology. That is, the concept that researchers should strive for objectivity and to capture a pre-existing reality [32]. It was therefore necessary for the lead researcher to devote additional time and effort to developing a ‘qualitative sensibility’, which is required for conducting research within a ‘Big Q’ qualitative paradigm [29,33]. In practice, this work involved continuous reflection of assumptions regarding knowledge production and analytical importance and interrogate where these assumptions may have been informed by the lead researcher’s predominantly positivist background, thus seeking to avoid ‘positivism creep’ [34].

### 2.2. Public and patient involvement

The study’s PPI collaborator (JP) is the Clare Island community representative on the Project team. In this instance our PPI representative played an equal and active part in shaping the research, contributing to the development of the research questions, recruitment, editing, and reviewing the final version of the study.

### 2.3. Study design

#### 2.3.1. Theoretical framework

Reflexive thematic analysis (RTA) was the methodological approach taken to guide the analysis of the data. RTA presents a theoretically flexible approach to qualitative analysis, facilitating the identification and in-depth interpretation of patterns and themes within a dataset [34]. This study aimed to explore the experiences of islanders engaging with novel technologies within the Home Health Project to support their health needs remotely. We also aimed to develop an understanding of the potential influences on the implementation of future iterations. Therefore, a flexible inductive and deductive approach with a contextualist epistemology underpinned the analysis, thus enabling the situating of the empirical findings within a theory-based framework (TDF). This approach facilitated the exploration of each participant’s subjective views on using the technologies while anchoring them within the shared reality of the study’s context [35].

#### 2.3.2. Ethical statement

This study received ethical approval from the Research Ethics Committee at the University of Galway. Prior to data collection, each participant was supplied with an information sheet and consent form. Participants were invited to review the information sheet detailing the study’s purpose and their involvement. Upon confirming their understanding and agreeing to participate verbally, formal written consent was collected.

### 2.4. Participant selection and participant information

Participants were recruited through purposive sampling. Individuals were eligible to participate if they were a member of the Clare Island community, over eighteen, and were participating in the broader Home Health Project. The PPI officer (JP) aided study recruitment by providing potential participants with a brief description of the study and a recruitment flyer containing the lead author’s contact information and a prompt to make contact if interested. In response, nine participants that met the inclusion criteria were invited to take part in the study. No participants dropped out of the study once they had agreed to participate. No reasons for refusing to participate were disclosed or recorded. The sample size was evaluated in the context of information power and deemed sufficient to address the study’s aims.

### 2.5. Data collection

Individual semi-structured interviews were conducted in-person in June 2023; interviews lasted approximately 45 minutes each. The interviews took place in the Home Health consultation pods located on Clare Island, Co. Mayo. An interview guide was informed by the theoretical domains framework (TDF), and the available extant literature related to the study (*S1 Appendix*). It was piloted with the Project PPI collaborator and refined. A flexible approach was taken to facilitate a conversational flow, allowing the lead author to adjust the questions depending on the individual experience of each participant [36]. The lead author (MK) conducted the interviews. Field notes were recorded after each interview. The interviews were audio-recorded with the participants’ consent, transcribed verbatim and imported into NVIVO 20 software for analysis.

### 2.6. Data analysis

This was a recursive process that moved between the six phases of the analytical approach outlined by Braun and Clarke [34]. To adhere to the fidelity of RTA when using both inductive and deductive coding methods, we did not incorporate incompatible positivist forms of thematic analysis (coding reliability TA; codebook approaches to TA) to achieve ‘reliable consensus’ [34]. Instead, the lead author (MK) and a second author (LR) engaged in reflexive shared-meaning discussions with the broader team to achieve richer interpretations of meaning within the dataset [37]. The lead author familiarised herself with the data by reading and re-reading the transcripts. Initial notes were taken at this stage to contextualise meaningful patterns identified across the dataset. Next, the data was coded at the semantic level using a ‘bottom-up’ inductive approach. The initial codes were participant-driven to capture both their insights about using novel technologies to support their health and their perspectives on the Home Health Project. As the Home Health Project is the first of its kind internationally, it was important to prioritise the participants’ insights before deductively coding the inductive codes to the relevant TDF domains. This was done to avoid making the data ‘fit’ within a theoretical framework [38]. The initial themes were generated by organising the inductive codes around core commonalities within the TDF domains and grouping them into themes. Leveraging reflexivity, the team engaged in sense-checking at each stage of the analysis ensuring congruency and coherence of the interpretations in light of the study aims. This iterative process led to the refinement and finalisation of the themes. The final themes, labelled as representative TDF domains, centre the participants’ insights while grounding them in theory to inform future iterations of the Home Health Project on Clare Island.

## 3. Results

A total of nine Clare Island residents participated in the study. Seven of the possible 14 TDF domains were supported by the empirical data as influences on engagement with the Project: Knowledge, Beliefs about capabilities, Optimism, Intentions, Environmental context and resources, Social influences and Emotion. Multiple extracts are presented in italics throughout to contextualise the research findings.

### 3.1. Knowledge

#### 3.1.1. Practical information

Prior to the initiation of the Project on the island, the islanders were aware of the Project but reported that initially, they had received little information about the purpose of the Project, stating “*in the beginning*, *we didn’t really know what it was”* [Participant 2]. However, as the Project team began to set up on the island, the islanders began to receive more information related to the overall Project, and regular updates to keep them informed. The more information made available to them resulted in a clearer understanding of the Project and what it involved: *“I think at the beginning*, *maybe a bit of apprehension*. *We didn’t… none of us really knew what it was about”* [Participant 1]. This journey of discovery was perceived to be expedited by having a local PPI (JP) representative on the research team. The PPI collaborator liaised with the participants, keeping them updated on the study objectives and providing support for any concerns that arose:

*“I think people seem happy enough now. And I’d say one of the reasons is probably because like Jack, he’s one of the islanders and you know, he’s very good at articulating what’s going on. So it lets us know what’s going on. So I think that’s very positive that we’ve got one of our own that can tell you exactly what’s going on you know”* [Participant 2].

Participants reported a gap in the information provided to them regarding the future direction of the Project, with many demonstrating an appetite to know more about the *“big picture”* and “*what they envisage the outcome to be”*:

*“I would like to know, like, say, this pilot Project here… If It’s successful…You know, if it happens, oh this is great, it’s worked. What would it mean for […] what will happen? Like [sic] what will the outcome of it be? Will it be that every little town might get, you know, a pod where they can go for cholesterol and heart checks? It would be just interesting to see, what do they envisage the outcome to be*” [Participant 2].

#### 3.1.2. Health information

Participants reported on the volume of health information supplied to them in the form of biofeedback. Physiological measurements such as cholesterol, BMI, height, weight, and blood pressure were taken and then provided to each participant as part of an initial consultation to establish a baseline. Participants valued having access to this information, describing it as *“good to know”* and “*interesting*”:

“*We all had our weight cholesterol and weight and all this done and height and everything… you know and our BMI is it? So like, it’s good to know…you’ve got you know, a base weight or base figures to start off with. So you’ve got something to improve on. So that’s good”* [Participant 2].

Participants also valued having an opportunity to clarify the health data with a healthcare provider to gain insight into their overall health and wellbeing, recounting that “*I began to understand a bit more about it myself”*.

Participants described the benefits of learning how to self-monitor their health using the technologies provided, reporting that it is *“great for seeing what you’re doing and what your activity levels”* and *“seeing how I slept”*. Participants described engaging with the technology regularly to track their health behaviours:

“*I look in the mornings to see how my sleep was on the tablet, because it does track your sleep and I was interested in seeing how I slept”* [Participant 7].

“*They’re (watches) great like [sic] for seeing*, *you know [sic] what you’re doing and what your activity levels are”* [Participant 5].

Participants described the benefits of being educated on the nutritional value of foods, learning how they would benefit from integrating them into their daily intake, and the impact that these changes, when implemented, would make on their over-all health and well-being:

“*The nutritionist came, and did the bloods and all those sort of things [sic] and looked at your diet or what you were eating. And then they recommended well, actually, you know, [sic] you need to cut back on this because of you know, the effects it has. But you need to increase on other things. You think oh, yes [sic] I found that really good”* [Participant 9].

*“The nutritional consultation was very good too*. *I’ve had that and I talked to someone about just like*, *[sic] you know different things with your diet that you can do*… *it was good*. *Very good”* [Participant 8].

### 3.2. Beliefs about capabilities

#### 3.2.1. Increased sense of autonomy

Participants described having an increased sense of autonomy over their healthcare as a result of engaging with the Home Health Project:

“*I started looking and realising that it’s not all about going to the doctor, getting the tablets and hopefully that solves your ills… That there’s actually…I have some control on my diet and my exercise. And maybe started thinking a different way, you know that I have some control of this myself like, you know”* [Participant 1].

For example, one participant recalled how engagement with the Project had allowed them to *“bring the control back a bit”* and to have the ability to take ownership over their own heath and related behaviours: *“part of the Project is your lifestyle*, *your health and how you take responsibility for it”* [Participant 9]. Participants also described their active involvement in the process as collaborators, whereby they had a sense of control over the direction of the Project which strengthened their active participation:

“*It seems a group Project that we’re all doing together [….] were all involved from the very beginning, input and everything”* [Participant 4].

#### 3.2.2. Using the technology

A mixed response was received from participants when asked about their confidence using the technologies provided by the Home Health Project. Some stated that they’re “*pretty confident*” and others stating that they’re “*not great on it*”. However, those less confident were motivated to keep using them describing how they were learning to adapt and expected that process to continue: *“we’ve kind of adapted to it [technology]*, *so I think we’ll just continue as time goes on*, *we’ll just continue to adapt to it”* [Participant 5]. Others once again described the usefulness of the PPI collaborator in assisting them with any technological difficulties they were having: *“I’m not great at it to be fair[…] so Jack helped me out*!*”* [Participant 7]. Regarding the usability of the self-monitoring technologies used by participants in this study (a wearable activity tracker and remote blood pressure monitor), participants described these devices as “*very straightforward*” and “*easy*” to use once they were shown how to navigate them. Difficulties that arose were due to reported issues with the reliability of the watches and the sensitivity of the in-built trackers. For example, one participant described an overestimation by the activity tracker of steps taken, while another reported an underestimation of steps taken:

“*I did find with this thing (smartwatch) when I go on the bus somewhere, when I’m driving… it thinks I’m walking, it thinks I’m going somewhere. So I’ve done all these kilometres and I’m thinking oh I’ve not walked anywhere yet today so…”* [Participant 2].

“*They weren’t very good watches that was the only bad thing about the whole thing*, *the watches are not reliable”* [Participant 2].

### 3.3. Optimism

The main finding identified within this domain encompasses the efficiency of the service provision both within their pre-existing healthcare service on the island and within the new resources available as part of the Project. Satisfaction with the care provided by the two local public health nurses was unanimous across all participants, with one individual describing the service as “*second to none*”. Participants also mentioned the emergency helicopter service, describing it as “*reassuring*” in the event of a health emergency. Participants displayed their strong faith in the healthcare services provided to them stating that “*there isn’t a better place to get a heart attack*” [Participant 3].

Participants went on to acknowledge the additional resources made available to them through the introduction of the Project, in particular, having access to specialised medical professionals at the touch of a button:

“*I suppose, without the Health Project, I wouldn’t have probably got to see a specialist like I did. And I’d just be carrying on with my blood pressure the way it is and I’d probably have heightened my risks down the line or in the short term, or whatever who knows like. So I feel it’s something that has become available to me that just wouldn’t have been there”* [Participant 1].

Participants mentioned the advantages of the Project as a “*preventative*” measure and the ability to “*identify a problem*” through more consistent remote monitoring that may have otherwise been missed:

*“Well, this is good, because it’s sort of preventative really, isn’t it? Like where you can see your triglyceride levels or cholesterol and heart rate or whatever. And think okay, well, we will try to improve that for the next six months”* [Participant 7].

*“They had discovered that my blood pressure is high*, *so I’ve been on a 24 hour monitor and the doctor here is on top of that now”* [Participant 6].

Time efficiency was another advantage of the Project frequently discussed by participants who described how the introduction of remote consultations is “*saving us trips up to Galway or Dublin or Castlebar*” (a journey from Clare Island to Galway takes approximately 2 hours and requires ferry and car travel). Participants also mentioned time efficiency of service provision, explaining that the Project “*saves waiting time in hospital*” as well as for accelerating the process of getting certain appointments that can usually take “*weeks*”.

“*If we have a problem we can just ring the doctor and you’re not waiting for weeks. Like I’ve just been to an osteopath, 8 weeks I was waiting for that. If you could just talk to someone and say oh well I’m feeling like that, they might say oh well to do these little exercises you know, just in the meantime”* [Participant 2].

Other conveniences of remote monitoring mentioned by participants include overcoming the issue of reliance on weather conditions and *“boats being cancelled”* when getting to appointments, as well as being able to speak with a health professional about something which *“doesn’t necessary require seeing them in person”*.

“*It’s marvelous to be able to talk to someone above in Galway hospital”* [Participant 1].

Participants also discussed their outlook on technology being integrated into healthcare in general. Many communicated that they are strongly in favour of this, stating it “*is the way forward”* and expressed their desire for a *“more wide-ranging approach”* in the future that encompasses all their healthcare needs:

“*The idea of taking a little pip of blood and putting it into a machine at the desk in front of you… and that’s gone off to Galway to get checked out, comes back immediately and you know where the hell you stand. I mean it’s just beyond wildest imagination”* [Participant 3].

However, along with this optimism, there was slight uncertainty amongst some participants regarding an over-reliance on technological approaches being used within healthcare. One individual explained that they suffer with eczema and that they are concerned a remote consultation would not suffice for conditions like this, mentioning *“you know if the quality* [of the remote consultation] *wasn’t great”*. Other participants talked about not wanting to be “*obsessed by what’s on the watch or the phone*” and mentioned that they are “*quite negative*” about technology development in general. Nonetheless, the reported convenience of the technologies was perceived to overcome the perceived challenges, in particular the use of technology to facilitate non-critical visits to the doctor, where a physical exam would not be required were seen to be a positive development:

“*I definitely think it’d be a good thing because, you know, like, often you might want to see a doctor and it might be just someone to ask if you’re worried about something. You don’t actually physically see them you just need to ask them”* [Participant 2].

In general, participants held an optimistic outlook on the Project as a whole. The community’s reaction to the introduction of this Project on the island was described as “*really positive*” with participants referring to it as “*the most extraordinary advance*” and a “*great initiative*”. Participants were optimistic when discussing the potential outcomes of the Project, mentioning that it could be “*the solution to overcrowding in hospitals during the day appointments anyway*” and that it “*could maybe save a life*, *or one of our lives down the road*” [Participant 1]. Participants generally reported high ambitions for the potential of the Project, however there were some concerns regarding its continuation. Participants stated: “*you’d hope that this won’t be something that happened on Clare Island and works and that this kind of pries [sic] to a halt*” and instead be “*the start of something that would continue on this island”* and *“be part of the solution*” [Participant 1].

### 3.4. Intentions

The optimistic perspectives participants held in relation to the Project were thought to increase their motivation and commitment to engage with the demands of the Project. Participants described their intention to “*contribute as much as you can*” to the Project and emphasised the importance of taking part in research like this Project, stating if “*people don’t put faith in things we won’t learn”* [Participant 1]. The perceived health benefits that the Project offered to participants was a driver of their motivation to engage with the research and their intention to implement and sustain the health changes required to optimise their overall health and well-being:

*“I think well anything like this is really positive for your health. My view at the beginning was, well, if you don’t do this, if you don’t participate in this… well, what does that say about your view on your own health? Because you know the Project is there to help you, that’s what it’s designed to do. It’s not designed for any other reason”* [Participant 9].

Participants also revealed their intentions to take action and improve their health since partaking in the Project. Participants discussed their health-related goals within the Project, such as “*to be more conscious of my health and do a bit more exercise or work on it*” [Participant 7]. Another participant mentioned how the Project has supplied them with “*base figures to start off with*” and their intention to “*improve on*” these. Participants have made conscious decisions to increase their physical activity by “*always trying to get to the 10*,*000 steps*” on their tracker, as well as, making an effort to improve their eating habits by “*eating more greens*” and “*reducing processed meats*” consumption.

“*You’re constantly aware of, you know [sic], improving yourself and being aware of ‘suppose[sic] like, my cholesterol was up at seven/eight. So I know it has come down. So I’m very much aware of trying to eat things that might lower that”* [Participant 2].

### 3.5. Environmental context and resources

In general, the participants reported that they are beholden to the local weather conditions on Clare Island on any given day, which can present a challenge when getting to appointments on the mainland, as if the weather is poor, the ferry service to and from the mainland does not operate:

*“I suppose the one thing I’ll say about appointments in hospital like it was, if I get a call in December or January, and the weather was bad, I cannot say I’m going to be there. I hope that I can be there. But you know, to this day that hasn’t changed because if the swells are bad then you maybe have to cancel that appointment and wait again”* [Participant 1].

This environmental barrier results in islanders having to take alternative measures to be able to attend appointments, which can come with additional burdens that incur time-related and financial costs:

“*You either take that chance get up in the morning and there may or may not be boat, I suppose. I’m especially talking about the winter months because that’s when you get most of the swells and wind and whatever. But yeah, the other option is having to go out days before and you know, food, accommodation and all that extra cost for maybe a ten minute appointment and get back again”* [Participant 1].

Restricted access to the mainland not only affects attending medical appointments but also their ability to obtain fresh food produce; participants explained that “*you mightn’t have the same choice of fresh fruit and vegetables*” on an island, and that if they cannot get to the mainland to restock, that their “*diet can kind of go downhill*, *you can go back into the old habits of the quickest or easiest thing to cook*” [Participant 1]. Participants also described the effects of the isolated environment on their personal lives, with some mentioning a lack of social activities on the island. They described how there “*isn’t any social element around things*” and stated that there is a community centre but “*it’s not consistent*”. One individual made a comparison to towns on the mainland, saying “*there’s plays and there’s different things that you can go to*, *cinemas and all of that*” and suggested that “*book clubs or even low impact exercise classes*” could be a good addition to the island community, emphasising that it “*definitely does help with mental health*” [Participant 4]. It is clear that the introduction of more social activities is a necessity for some participants as they state that “*it’s not doing me any good anyway isolated or on my own*” and how it influences them to engage in negative health behaviours: “*I used to go to the AA meetings*, *I would tend to start drinking*” [Participant 4].

### 3.6. Social influences

Participants described the small close-knit community that exists on Clare Island, explaining that “*everybody knows everybody*”. Participants discussed the social support that exists within a close-knit community like Clare Island, explaining that “*you meet someone out walking*, *and you’re like*, *oh*, *you’re getting your steps in*, *because everyone has the watch you know*” [Participant 8]. Although participants were mostly positive about everyone being well acquainted on the island, some did mention difficulties with maintaining anonymity within the Project:

“*You kind of bump into the next person coming out of here (the pods) and you’d kind of know that way that they’re doing the same as yourself*” [Participant 1].

Participants again highlighted the advantage of having a local PPI collaborator in the Project: “*I think that’s very positive*, *that we’ve got one of our own that can tell you exactly what’s going on you know*” [Participant 2]. They emphasised the importance of having local people involved, with one participant stating that “*they have assisted because of their understanding as well of the island*. *That would be critical I would have thought of to getting it off the ground*” [Participant 9]. Participants considered themselves to be well supported by the Project team for the most part and mentioned support is available if they need it, explaining that *“they’re very accessible to us if we need them which is great”* [Participant 4]. However, some participants did describe a decrease in momentum in comparison to the beginning of the Project, stating that “*we’re getting very good advice but it’s like I’m on my own a bit then again in a sense*” [Participant 1].

#### 3.6.1. Increases social interactions

Participants described how, as a community on the island, they have begun *“talking more about health*” since the Project began:

“*People that I never would dream of would have blood pressure say ah yeah sure I have blood pressure and you know you kinda wind up taking a bit more about things”* [Participant 1].

There were active interactions between the islanders and the Project team via a WhatsApp group. This provided an additional medium for the participants to query any concerns that arise, which was seen as particularly beneficial for its convenience in lieu of having to go online for small queries:

“*These interactions are excellent and for you to be here it’s great as well rather than doing it online or whatever. And do be truthful I think if I was asked to do it online I don’t know whether I would or not”* [Participant 4].

The need for social interaction was discussed by many participants who emphasised the importance of face-to-face interaction, stating that *“you need the personal interaction”*. Participants were mostly positive about the introduction of technology but stressed the importance of maintaining in-person interactions with one participant explaining that *“you can’t substitute personal care*. *You can’t substitute personal relationships*, *face to face”* [Participant 5].

### 3.7. Emotion

Participants in the study described a range of different emotions in response to the implementation of the Project. Some described *“a bit of apprehension”* at the beginning and explained that they *“didn’t really know what it was”*. One participant also described their first experience of talking to a screen as *“a little strange”*. Another factor contributing to the apprehension surrounding the Project is a fear of losing aspects of their pre-existing healthcare service. Participants described concerns that the technology being introduced as part of this Project would “*replace”* their current system and that *“there wouldn’t be a nurse on the island”* anymore. Participants were clear that technology should enhance the current service as they *“wouldn’t like technology to come and just replace what we have already”* [Participant 2].

Concerns were raised surrounding data protection in the study. Participants reported being apprehensive about a lack of knowledge of where their health information will be stored and who will have access to it:

*“*[Participants] *would be concerned about where this information goes, especially to do with things being hacked”* [Participant 9].

When asked about potential future advancements within the Project such as a home telepresence system being introduced, participants expressed apprehension, explaining that they feel *“less keen on that*” but some individuals did recognise the potential benefits of such an intervention for an “*elderly relative*” to *“give them a bit longer”* at home instead of residing in a nursing home.

## 4. Discussion

### 4.1. Principal findings

The current study provides insight into the early-stage implementation of the Home Health Project by documenting the real-life experiences of participants within this trial, as well as their perspectives on the introduction of technology into healthcare in general. The Theoretical Domains Framework (TDF) was applied to systematically identify factors that potentially influence participants’ engagement with the Project. A total of seven domains were identified as influential on behaviour within the early-stage implementation of this trial: knowledge, beliefs about capabilities, optimism, intentions, environmental context and resources, social influences, and emotion.

Perspectives on the Project were largely positive, with participants mentioning increased health literacy, empowerment, efficiency of services provided and good support as the main benefits of Project participation thus far. Some issues discussed by participants included faulty technology and the need for more personalised advice and feedback. There was a great sense of optimism for the successful implementation and impact of the Project. However, some participants expressed concerns around the general use of technology in healthcare such as an overdependency on devices, perceived risks around data protection, lack of digital literacy, a need for personalised care and a fear of losing existing healthcare services on the island. Participants also expressed some apprehension regarding the prospect of the introduction of even more technological resources within Project in the future.

The provision of knowledge was highlighted in the findings as a large benefit of the Home Health Project, with participants receiving an abundance of information in the form of biofeedback, nutritional guidance, and activity tracking. Participants mentioned the benefit of increased health literacy and expressed their interest in learning about their health. A previous review exploring factors that affect patient and public involvement in digital health interventions similarly found that people enjoyed gaining more knowledge about their health behaviours [39]. Participants suggested recording in-person information sessions within the Project to allow for those that are busy to listen in their own time and enable individuals to listen back to the information if they require. It has been evident from previous research that conflicting priorities and busy personal lives affect one’s engagement with interventions [39]. Proliferation of health-related knowledge facilitates health related behaviour change and acts as a catalyst for several other crucial factors, including patient engagement and empowerment [40].

Empowerment refers to “*an enabling process or an outcome of a process involving a shift in the balance of power”* [41] and represents another key theme that was generated within the ‘belief about capabilities’ domain. Patient empowerment is associated with increased knowledge, skills, self-awareness and confidence amongst patients to take an active role in maintaining their health [42]. This was reflected in the findings, as participants portrayed increased perceived capability for taking control in their own healthcare, with many describing how they have embraced the responsibility they hold within their healthcare since partaking in the trial. Empowerment is also positively associated with patient involvement in the form of self-care and shared decision making [40]. Similar findings were seen in this study, as participants discussed being more involved their healthcare and setting more health-related goals since their involvement in the Project, such as increasing their physical activity and improving their eating habits.

Setting of health-related goals is a theme that was generated within the ‘intentions’ domain of the TDF. Personal motivation to manage and improve your health is fundamental for engagement in telehealth interventions [43]. Participants described how self-monitoring with the use of digital devices caused them to be more aware of their bodily functions and influenced them to make more conscious health related decisions. This finding is consistent with previous research which reports that technological interventions are effective in maintaining an individual’s motivation to prevent the development of disease, lose weight or be physically active [39]. Participants were largely positive about gaining more control and improving their health literacy through the introduction of the Project. It has been reported within previous healthcare studies that participants appreciate the opportunity to gain more control over aspects such as diet and exercise or where applicable, even self-manage chronic health conditions [39].

Personalisation of health interventions has previously been identified as one of the main facilitators of patient engagement [42] and was a component the participants of this study described as lacking within the Home Health Project. Participants discussed their need for a more personalised approach within the Project, such as dietary guidelines tailored to their individual needs and individualised feedback. Personalised digital health interventions have been associated with optimised healthcare and health status amongst patients [44]. However, participants did acknowledge the active role they play within the Project and the opportunity to give their input if they desire. Person-centred care reflects individualisation of care and is associated with enhanced engagement in health interventions [42]. The Home Health Project adopted a community-based approach with a large focus on Public and Patient Involvement, and this reflected very positively in the findings. Findings under the ‘social’ TDF domain highlighted the importance of support within the Clare Island community. Having a pre-existing relationship with the core Home Health Project team was consistently referred to by participants as a huge advantage. Recommendations from trusted individuals and social support were identified as important for engagement within previous research based on digital health technologies, which found that those lacking such support failed to engage [45].

Technological capabilities were another recurring articulation within the findings, with each participant reporting a different level of digital literacy, mainly based on their previous experience using technology. The usability of a digital health intervention has been shown to affect participant engagement with that intervention [45]. Participants described the Home Health Project as nonburdensome and straight forward overall, however there was a consensus across all participants in this study that the wearable activity trackers were inaccurate and not fit for purpose. This represents a potential barrier of high importance to the uptake of this intervention within the Project.

Participants discussed their pre-existing healthcare system on Clare Island and satisfaction with the services already available to them was unanimous across all participants. However, remote island communities are presented with numerous challenges in accessing equitable healthcare comparative to in urban areas, with geographical restrictions and unpredictable weather conditions representing some of the challenges faced by islanders daily in accessing healthcare services [5]. Participants discussed how the Home Health Project is already beginning to ease these longstanding environmental barriers within their healthcare, reducing burdensome trips to the mainland for health-related appointments and saving them time overall.

### 4.2. Strengths & Limitations

A key strength of this study includes the exploratory approach, using qualitative methodology, to obtain compelling and valuable insights into participant’s unique lived experience engaging in the Home Health Project as well as their perspectives on the incorporation of technology within healthcare. Furthermore, the use of the Theoretical Domains Framework (TDF) assisted in identifying key influential factors that may impact on participants’ future engagement in the Project while providing robust theoretical backup.

However, although the use of the TDF provided structure within the analysis, it restricts the leeway for subjectivity when situating themes in a particular domain. Many codes generated from the dataset had some relevance in several different domains and assigning them to one domain restricted flexibility in comparison to solely using thematic analysis. Other limitations include the small sample size recruited in this study—although it is adequate in terms of information power [30]. Given that this is a novel Project, each participant portrayed unique experiences, and a larger sample size would allow for a more comprehensive exploration of these experiences and individual needs.

### 4.3. Future directions

The incorporation of the more extensive Behaviour Change Wheel framework in combination with this TDF analysis would contribute to a more comprehensive behavioural assessment. This would allow for the behavioural influences identified in this study to be mapped on to the COM-B model of behaviour as well as specific intervention functions in relation to the TDF. This would benefit researchers on the Home Health Project by providing more clarity and identifying specific plans of action to remedy issues noted by participants in this study.

## 5. Conclusion

The present study sought to explore the personal experiences and perspectives of participants in the Clare Island Home Health Project, an ongoing trial involving the introduction of digital technology to the delivery of healthcare to individuals living on Clare Island, a remote community in the west of Ireland. Semi-structured interviews were conducted to collect qualitative data, and a reflexive thematic analysis approach was taken. The data was coded inductively initially, and then codes were deductively mapped on to the Theoretical Domains Framework (TDF) to identify determinants of engagement with the Home Health Project. A total of seven domains were identified as influential to behaviour within the early-stage implementation of this trial: knowledge, beliefs about capabilities, optimism, intentions, environmental context and resources, social influences, and emotion. The main benefits of participation in the Project identified within the dataset include increased health literacy, empowerment, efficiency of services and support, whereas faulty technology and a need for greater personalisation of advice and feedback were cited as barriers by the participants. The findings of this study have implications for the development and implementation of digital health behaviour change interventions and demonstrate the potential for digital health to make healthcare more accessible to all.

## Supporting information

S1 Appendix Interview Protocol. (DOCX)

S2 Appendix COREQ Checklist. (DOCX)

## References

[1] Inglis SC, Clark RA, McAlister FA, Ball J, Lewinter C, Cullington D, Stewart S, Cleland JG. Structured telephone support or telemonitoring programmes for patients with chronic heart failure. Cochrane database of systematic reviews. 2010(8). doi: 10.1002/14651858.CD007228.pub2 20687083

[2] Lupton D. Quantifying the body: monitoring and measuring health in the age of mHealth technologies. Critical public health. 2013 Dec 1;23(4):393–403.

[3] Arden NS, Fisher AC, Tyner K, Lawrence XY, Lee SL, Kopcha M. Industry 4.0 for pharmaceutical manufacturing: Preparing for the smart factories of the future. International Journal of Pharmaceutics. 2021 Jun 1;602:120554. doi: 10.1016/j.ijpharm.2021.120554 33794326

[4] Awad A, Trenfield SJ, Pollard TD, Ong JJ, Elbadawi M, McCoubrey LE, Goyanes A, Gaisford S, Basit AW. Connected healthcare: Improving patient care using digital health technologies. Advanced Drug Delivery Reviews. 2021 Nov 1;178:113958. doi: 10.1016/j.addr.2021.113958 34478781

[5] Murray A, Lineen-Curtis N, Worlikar H, O’Keeffe D. Clare Island Digital Health Project-using technology to enable health for all. Rural and Remote Health. 2023 Jan 10;23(1):8175. doi: 10.22605/RRH8175 36802941

[6] Antezana FS, Chollat-Traquet CM, Yach D. Health for all in the 21st century. World health statistics quarterly (Rapport trimestriel de statistiques sanitaires mondiales 1998; 51 (1): 3–6). 1998. 9675803

[7] National Rural Health Alliance. Inquiry into the My Health Record System: Senate Committee on Community Affairs. National Rural Health Alliance 2018; 9.

[8] Lipworth W, Taylor N, Braithwaite J. Can the theoretical domains framework account for the implementation of clinical quality interventions?. BMC health services research. 2013 Dec;13:1–3. 24359085 10.1186/1472-6963-13-530 PMC3901331

[9] Craig P, Dieppe P, Macintyre S, Michie S, Nazareth I, Petticrew M. Developing and evaluating complex interventions: the new Medical Research Council guidance. Bmj. 2008 Sep 29;337. doi: 10.1136/bmj.a1655 18824488 PMC2769032

[10] Davies P, Walker AE, Grimshaw JM. A systematic review of the use of theory in the design of guideline dissemination and implementation strategies and interpretation of the results of rigorous evaluations. Implementation science. 2010 Dec;5:1–6. 20181130 10.1186/1748-5908-5-14 PMC2832624

[11] Colquhoun HL, Brehaut JC, Sales A, Ivers N, Grimshaw J, Michie S, Carroll K, Chalifoux M, Eva KW. A systematic review of the use of theory in randomized controlled trials of audit and feedback. Implementation Science. 2013 Dec;8:1–8. 23759034 10.1186/1748-5908-8-66 PMC3702512

[12] Atkins L, Francis J, Islam R, O’Connor D, Patey A, Ivers N, Foy R, Duncan EM, Colquhoun H, Grimshaw JM, Lawton R. A guide to using the Theoretical Domains Framework of behaviour change to investigate implementation problems. Implementation science. 2017 Dec;12:1–8. 28637486 10.1186/s13012-017-0605-9 PMC5480145

[13] Mumtaz H, Riaz MH, Wajid H, Saqib M, Zeeshan MH, Khan SE, Chauhan YR, Sohail H, Vohra LI. Current challenges and potential solutions to the use of digital health technologies in evidence generation: a narrative review. Frontiers in Digital Health. 2023 Sep 28;5:1203945. doi: 10.3389/fdgth.2023.1203945 37840685 PMC10568450

[14] Taylor N, Conner M, Lawton R. The impact of theory on the effectiveness of worksite physical activity interventions: a meta-analysis and meta-regression. Health Psychology Review. 2012 Mar 1;6(1):33–73.

[15] Webb T, Joseph J, Yardley L, Michie S. Using the internet to promote health behavior change: a systematic review and meta-analysis of the impact of theoretical basis, use of behavior change techniques, and mode of delivery on efficacy. Journal of medical Internet research. 2010 Feb 17;12(1):e1376. doi: 10.2196/jmir.1376 20164043 PMC2836773

[16] Finne E, Glausch M, Exner AK, Sauzet O, Stoelzel F, Seidel N. Behavior change techniques for increasing physical activity in cancer survivors: a systematic review and meta-analysis of randomized controlled trials. Cancer management and research. 2018 Oct 30:5125–43. doi: 10.2147/CMAR.S170064 30464612 PMC6215922

[17] Michie S, Carey RN, Johnston M, Rothman AJ, De Bruin M, Kelly MP, Connell LE. From theory-inspired to theory-based interventions: a protocol for developing and testing a methodology for linking behaviour change techniques to theoretical mechanisms of action. Annals of behavioral medicine. 2018 Jun;52(6):501–12. doi: 10.1007/s12160-016-9816-6 27401001 PMC6367898

[18] Michie S, Johnston M, Abraham C, Lawton R, Parker D, Walker A. Making psychological theory useful for implementing evidence based practice: a consensus approach. BMJ quality & safety. 2005 Feb 1;14(1):26–33. doi: 10.1136/qshc.2004.011155 15692000 PMC1743963

[19] Michie S, Van Stralen MM, West R. The behaviour change wheel: a new method for characterising and designing behaviour change interventions. Implementation science. 2011 Dec;6:1–2. 21513547 10.1186/1748-5908-6-42 PMC3096582

[20] Michie S, Atkins L, West R. The behaviour change wheel. A guide to designing interventions. 1st ed. Great Britain: Silverback Publishing. 2014;1003:1010.

[21] Timlin D, McCormack JM, Simpson EE. Using the COM-B model to identify barriers and facilitators towards adoption of a diet associated with cognitive function (MIND diet). Public health nutrition. 2021 May;24(7):1657–70. doi: 10.1017/S1368980020001445 32799963 PMC8094434

[22] Martin R, Murtagh EM. An intervention to improve the physical activity levels of children: Design and rationale of the ‘Active Classrooms’ cluster randomised controlled trial. Contemporary clinical trials. 2015 Mar 1;41:180–91. doi: 10.1016/j.cct.2015.01.019 25657052

[23] Cowdell F, Dyson J. How is the theoretical domains framework applied to developing health behaviour interventions? A systematic search and narrative synthesis. BMC public health. 2019 Dec;19:1–0. 31455327 10.1186/s12889-019-7442-5 PMC6712870

[24] McEachan RR, Santorelli G, Bryant M, Sahota P, Farrar D, Small N, Akhtar S, Sargent J, Barber SE, Taylor N, Richardson G. The HAPPY (Healthy and Active Parenting Programmme for early Years) feasibility randomised control trial: acceptability and feasibility of an intervention to reduce infant obesity. BMC public health. 2016 Dec;16:1–5. 26931491 10.1186/s12889-016-2861-z PMC4774160

[25] Taylor NJ, Sahota P, Sargent J, Barber S, Loach J, Louch G, Wright J. Using intervention mapping to develop a culturally appropriate intervention to prevent childhood obesity: the HAPPY (Healthy and Active Parenting Programme for Early Years) study. International Journal of Behavioral Nutrition and Physical Activity. 2013 Dec;10:1–6. 24373301 10.1186/1479-5868-10-142 PMC3895739

[26] Van Agteren JE, Lawn S, Bonevski B, Smith BJ. Kick. it: the development of an evidence-based smoking cessation smartphone app. Translational behavioral medicine. 2018 Apr;8(2):243–67. doi: 10.1093/tbm/ibx031 29447386

[27] Connolly C, Hernon O, Carr P, Worlikar H, McCabe I, Doran J, Walsh JC, Simpkin AJ, O’Keeffe DT. Artificial Intelligence in Interprofessional Healthcare Practice Education–Insights from the Home Health Project, an Exemplar for Change. Computers in the Schools. 2023 Oct 2;40(4):412–29.

[28] Tong A, Sainsbury P, Craig J. Consolidated criteria for reporting qualitative research (COREQ): a 32-item checklist for interviews and focus groups. International journal for quality in health care. 2007 Dec 1;19(6):349–57. doi: 10.1093/intqhc/mzm042 17872937

[29] Braun V, Clarke V. Reflecting on reflexive thematic analysis. Qualitative research in sport, exercise and health. 2019 Aug 8;11(4):589–97.

[30] Malterud K, Siersma VD, Guassora AD. Sample size in qualitative interview studies: guided by information power. Qualitative health research. 2016 Nov;26(13):1753–60. doi: 10.1177/1049732315617444 26613970

[31] Braun V, Clarke V. Toward good practice in thematic analysis: Avoiding common problems and be (com) ing a knowing researcher. International journal of transgender health. 2023 Jan 25;24(1):1–6. doi: 10.1080/26895269.2022.2129597 36713144 PMC9879167

[32] Burman E. Minding the gap: Positivism, psychology, and the politics of qualitative methods. Journal of Social Issues. 1997 Jan;53(4):785–801.

[33] Ashworth P. Conceptual foundations of qualitative psychology. Qualitative psychology: A practical guide to research methods. 2008;2:4–25.

[34] Braun V. Clarke V. Thematic analysis: A practical guide. Los Angeles: Sage; 2022.

[35] Braun V, Clarke V. Using thematic analysis in psychology. Qualitative research in psychology. 2006 Jan 1;3(2):77–101.

[36] McGowan LJ, Powell R, French DP. How can use of the Theoretical Domains Framework be optimized in qualitative research? A rapid systematic review. British Journal of Health Psychology. 2020 Sep;25(3):677–94. doi: 10.1111/bjhp.12437 32558289

[37] Paulus TM, Woodside M, Ziegler MF. “I tell you, it’sa journey, isn’t it?” Understanding collaborative meaning making in qualitative research. Qualitative Inquiry. 2010 Dec;16(10):852–62.

[38] Garvey CM, Jones R. Is there a place for theoretical frameworks in qualitative research?. International Journal of Qualitative Methods. 2021 Feb 26;20:1609406920987959.

[39] O’Connor S, Hanlon P, O’donnell CA, Garcia S, Glanville J, Mair FS. Understanding factors affecting patient and public engagement and recruitment to digital health interventions: a systematic review of qualitative studies. BMC medical informatics and decision making. 2016 Dec;16:1–5. 27630020 10.1186/s12911-016-0359-3 PMC5024516

[40] Hickmann E, Richter P, Schlieter H. All together now–patient engagement, patient empowerment, and associated terms in personal healthcare. BMC health services research. 2022 Sep 2;22(1):1116. doi: 10.1186/s12913-022-08501-5 36056354 PMC9440506

[41] Cerezo PG, Juvé-Udina ME, Delgado-Hito P. Del concepto de empoderamiento del paciente a los instrumentos de medida: una revisión integrativa. Revista da Escola de Enfermagem da USP. 2016 Jul;50:0667–74.

[42] Higgins T, Larson E, Schnall R. Unraveling the meaning of patient engagement: a concept analysis. Patient education and counseling. 2017 Jan 1;100(1):30–6. doi: 10.1016/j.pec.2016.09.002 27665500

[43] Miyamoto S, Henderson S, Young H, Ward D, Santillan V. Recruiting rural participants for a telehealth intervention on diabetes Self-Management. The Journal of Rural Health. 2013 Jan;29(1):69–77. doi: 10.1111/j.1748-0361.2012.00443.x 23289657 PMC3539245

[44] Maeckelberghe E, Zdunek K, Marceglia S, Farsides B, Rigby M. The ethical challenges of personalized digital health. Frontiers in Medicine. 2023 Jun 19;10:1123863. doi: 10.3389/fmed.2023.1123863 37404804 PMC10316710

[45] Bardus M, Blake H, Lloyd S, Suzanne Suggs L. Reasons for participating and not participating in a e-health workplace physical activity intervention: A qualitative analysis. International Journal of Workplace Health Management. 2014 Nov 4;7(4):229–46.

