# Supplementary material for: A qualitative exploration of participants’ perspectives and experiences of novel digital health infrastructure to enhance patient care in remote communities within the Home Health Project
Source: PLOS Digit Health. 2024 Nov 1;3(11):e0000600. doi: 10.1371/journal.pdig.0000600 (PMC11530050; doi:10.1371/journal.pdig.0000600)
Supplement: S1 Appendix — (DOCX) [file pdig.0000600.s001.docx]

**S1 Appendix: Interview Protocol**

| **Greeting and introduction** | Hello, my name is Madeleine Kearney. I would like to thank you for agreeing to participate in this interview. Your time, and the information you share with me is very much appreciated and will contribute greatly to my research |  |
| --- | --- | --- |
| **Interview Topic Guide** | **Overarching Question(s)** |  |
| General experience of healthcare on the island | Could you describe your healthcare on Clare Island?   - If you needed to see a doctor? - If there was an emergency? - If you had a hospital appointment to attend? |  |
|  |  |  |
| Experience of the Home Health Project | Could you discuss your understanding of the Home Health Project?    Explain your involvement in the study thus far?  Have you used any devices as part of the project?   - Activity tracker/ blood pressure monitor   ..How did you find this?    Have you experienced a remote/ initial consultation nutritional consultation? How did you find this?    Has it caused any changes in your healthcare so far?    How have you found your experience in the project thus far?    Has this intervention influenced changes in your day to day life? |  |
|  |  |  |
|  |  |  |
| Perspectives on the Home Health Project | What are your thoughts on the Home health project?  Potential outcomes of this project?  (positives/benefits)  Views on the integration of technology into healthcare in general?  View on potential future interventions? (telepresence) | |
| Usability of technology | Do you have experience using technology before this project?  How would you describe you level of ability using technology?  Do you feel adequately educated on the technology you are required to use as part of this intervention?  How have you found using the devices as part of this project? |  |
|  |  |  |
| Support Elements            Suggestions for the project | Do you feel satisfied with the level of support you are receiving within the project?  If you had an issue within the project, what would you do?  Do you discuss your involvement in the project with other members of the community?  Do you have any suggestions for things you would like incorporated more into the project?  Needs that aren’t met?  Any elements you have found good/bad |  |
| **Closing the interview** | Is there anything else that you would like to add?  Thank you for taking time to contribute to this research. I really appreciate you sharing your experiences with me. Your contributions will be invaluable within my research process. |  |
