# Supplementary material for: A qualitative exploration of participants’ perspectives and experiences of novel digital health infrastructure to enhance patient care in remote communities within the Home Health Project
Source: PLOS Digit Health. 2024 Nov 1;3(11):e0000600. doi: 10.1371/journal.pdig.0000600 (PMC11530050; doi:10.1371/journal.pdig.0000600)
Supplement: S2 Appendix — (DOCX) [file pdig.0000600.s002.docx]

**S2 Appendix: COREQ Checklist**
